# Development and Validation of a Quantitative, High-Throughput, Fluorescent-Based Bioassay to Detect *Schistosoma* Viability

**DOI:** 10.1371/journal.pntd.0000759

**Published:** 2010-07-27

**Authors:** Emily Peak, Iain W. Chalmers, Karl F. Hoffmann

**Affiliations:** Institute of Biological, Environmental and Rural Sciences, Aberystwyth University, Aberystwyth, United Kingdom; Swiss Tropical Institute, Switzerland

## Abstract

**Background:**

Schistosomiasis, caused by infection with the blood fluke *Schistosoma*, is responsible for greater than 200,000 human deaths per annum. Objective high-throughput screens for detecting novel anti-schistosomal targets will drive ‘genome to drug’ lead translational science at an unprecedented rate. Current methods for detecting schistosome viability rely on qualitative microscopic criteria, which require an understanding of parasite morphology, and most importantly, must be subjectively interpreted. These limitations, in the current state of the art, have significantly impeded progress into whole schistosome screening for next generation chemotherapies.

**Methodology/Principal Findings:**

We present here a microtiter plate-based method for reproducibly detecting schistosomula viability that takes advantage of the differential uptake of fluorophores (propidium iodide and fluorescein diacetate) by living organisms. We validate this high-throughput system in detecting schistosomula viability using auranofin (a known inhibitor of thioredoxin glutathione reductase), praziquantel and a range of small compounds with previously-described (gambogic acid, sodium salinomycin, ethinyl estradiol, fluoxetidine hydrochloride, miconazole nitrate, chlorpromazine hydrochloride, amphotericin b, niclosamide) or suggested (bepridil, ciclopirox, rescinnamine, flucytosine, vinblastine and carbidopa) anti-schistosomal activities. This developed method is sensitive (200 schistosomula/well can be assayed), relevant to industrial (384-well microtiter plate compatibility) and academic (96-well microtiter plate compatibility) settings, translatable to functional genomics screens and drug assays, does not require *a priori* knowledge of schistosome biology and is quantitative.

**Conclusions/Significance:**

The wide-scale application of this fluorescence-based bioassay will greatly accelerate the objective identification of novel therapeutic lead targets/compounds to combat schistosomiasis. Adapting this bioassay for use with other parasitic worm species further offers an opportunity for great strides to be made against additional neglected tropical diseases of biomedical and veterinary importance.

## Author Summary

With only one effective drug, praziquantel, currently used to treat most worldwide cases of schistosomiasis, there exists a pressing need to identify alternative anthelmintics before the development of praziquantel-resistant schistosomes removes our ability to combat this neglected tropical disease. At present, the most widely adopted methodology used to identify promising new anti-schistosome compounds relies on time consuming and subjective microscopic examination of parasite viability in response to *in vitro* schistosome/compound co-culturing. In our continued effort to identify novel drug and vaccine targets, we detail a dual-fluorescence bioassay that can objectively be used for assessing *Schistosoma mansoni* schistosomula viability in a medium or high- throughput manner to suit either academic or industrial settings. The described methodology replaces subjectivity with sensitivity and provides an enabling technology useful for rapid *in vitro* screens of both natural and synthetic compound libraries. It is expected that results obtained from these quantifiable *in vitro* screens would prioritize the most effective anti-schistosomal compounds for follow-up *in vivo* experimentation. This highly-adaptable dual-fluorescence bioassay could be integrated with other methods for measuring schistosome phenotype and, together, be used to greatly accelerate our search for novel anthelmintics.

## Introduction

Infection with the parasitic trematode *Schistosoma mansoni* causes a wide range of quantifiable clinical pathologies [1], which collectively lead to the death of approximately 200,000 individuals/annum [2]. Recent ‘first pass’ description of this parasite's genome [3], as well as multiple reports describing the utilization of numerous functional genomics tools (e.g. [4], [5]), have now provided the technological framework for a renaissance in drug target and vaccine discovery research [6]. A major bottleneck in converting schistosome phenotypic discovery into applied therapeutic products, however, is the lack of appropriate methods for quantifying, in a high-throughput manner, individual gene function or small compound effect on parasite survival. Therefore, development of reproducible, non-subjective methods for high-throughput screening of parasite viability would present the schistosome community with a tangible opportunity to translate genomic and functional genomics information into therapeutic strategies to combat schistosomiasis.

Current methods utilized to assess schistosome and other trematode viability have recently been reviewed [7]. All involve microscopic techniques where the experimenter manipulates the parasite *in vitro* and assesses the effect of such manipulation by bright-field examination of morphology. This technique has been employed in immunological studies [8], RNA interference (RNAi) assays [9], drug screening protocols [9], [10] and general manipulations of parasite development [11]. Criteria used to assess schistosome viability in these investigations include intracellular granularity, parasite movement, parasite shape alterations and uptake of various vital dyes (e.g. methylene blue or toluidine blue). The subjective nature of these various and time-consuming measurement indices indicate that inter-laboratory estimates of schistosome viability in response to *in vitro* manipulation will be quite variable, resulting in a lack of uniform reporting within the community.

We report here on an improved methodology to objectively detect parasite survival during *in vitro* culture. The fundamental principle of this assay is derived from the differential membrane permeability of two well-known dyes, fluorescein diacetate (FDA, an esterase substrate) and propidium iodide (PI, a DNA intercalating agent). Based on previously described uses of these two dyes [12], it was anticipated that FDA would cross the membranes of living cells (within living schistosomes) and be converted into highly-fluorescent and charged fluorescein (which cannot readily leave live cells) by parasite esterase activity. In contrast to FDA, PI would not be able to cross the membranes of living parasites and could only stain nucleic acids if there was a breach in membrane permeability (due to parasite death). In dead parasites, we hypothesized that PI, and not FDA, would preferentially stain the assayed sample and, thus, changes in both dye's fluorescent intensities would be indicative of schistosome viability, which could subsequently be quantified by a plate reader, fluorescent microscope or any device equipped to measure fluorescence. This single property (simultaneous detection of both PI and FDA measures) has allowed us to develop a fluorescence-based, microtiter-plate bioassay to improve detection of schistosome viability, which is high-throughput (96- and 384-well capacity), quantitative and provides objective readouts (fluorescence intensity units) of parasite survival during *in vitro* culture.

Using this flexible bioassay, we demonstrate its versatility in detecting schistosome survival in response to thioredoxin glutathione reductase (TGR) inhibition [9]. Furthermore, we adopt this assay to provide quantitative estimates of schistosome viability in the presence of recently identified small compounds with previously described [10] and unknown [3] chemotherapeutic activities. Implementation of this novel screening platform by academia and industrial stakeholders will enable inter-laboratory comparisons of *in vitro* parasite manipulations to be routinely and quickly performed, vastly accelerating the search for novel anti-schistosomal lead targets.

## Methods

### Schistosomula preparation and culturing

*Schistosoma mansoni* (Puerto Rican strain) infected *Biomphalaria glabrata* snails were provided by Fred Lewis (Biomedical Research Institute, Rockville, MD, USA). Cercariae were shed from infected snails by exposure to light (60 min at room temperature, RT) and subsequently converted to schistosomula by mechanical transformation [13]. Schistosomula were purified away from cercarial tails by centrifugation through a 60% percoll gradient [14]. Microscope examination was used to assess the quantity and quality of purified schistosomula. Schistosomula were cultured at 37°C in T25 tissue culture flasks containing 9 ml DMEM (Dulbecco's Modified Eagle Medium, Sigma-Aldrich), lacking phenol red but containing 4500 mg/l glucose, supplemented with 10% foetal calf serum, 2 mM *L*-glutamine, 200 U/ml penicillin, 200 µg/ml streptomycin (all Sigma-Aldrich) in an atmosphere of 5% CO_2_ for 24 hr before any further experimental manipulations proceeded. Negligible parasite death occurred in this media during the 24 hr culturing period. Following this, schistosomula were aliquoted into black-sided, flat-bottom (optically clear), 96-well microtiter plates (Fisher Scientific) in 200 µl media or black-sided, flat-bottom (optically clear), 384-well microtiter plates (Matrix) in 40 µl media.

Heat killed schistosomula were also prepared by incubating the 24 hr cultivated parasites at 65°C for 10 min. These dead schistosomula were allowed to cool to 37°C before being used in subsequent experiments.

### Microscopy

Live and heat killed schistosomula stained with optimal concentrations (empirically derived from [12]) of propidium iodide (PI, 2.0 µg/ml; Sigma-Aldrich), fluorescein diacetate (FDA, 0.5 µg/ml; Sigma-Aldrich) or both fluorophores were visualized at ×100 magnification using a Leica Axioplan microscope equipped with FITC (494 excitation) and Rhodamine (536 excitation) filters and a mercury vapor light source. A Hamamatsu CA74295 camera with Wasabi Version 1.4 software was used to capture photographic images of stained schistosomula.

Schistosomula, co-incubated with test compounds, were fluorescently visualized as above or unstained at ×100 magnification using an Olympus CK2 inverted microscope equipped with a stage extension plate and specimen holder for handling microtiter plates. A Kodak EasyShare DX7440 digital camera was used to capture images of unstained schistosomula.

### Compound storage, handling and schistosomula screening

All compounds were purchased from Sigma-Aldrich and included: gambogic acid, sodium salinomycin, ethinyl estradiol, fluoxetidine hydrochloride, bepridil, ciclopirox, miconazole nitrate, chlorpromazine hydrochloride, amphotericin b, niclosamide, rescinnamine, flucytosine, vinblastine, carbidopa, praziquantel and auranofin.

Stock solutions of all compounds were made up at 1 mM in appropriate solvents (Dataset S1) and stored at −80°C. All compounds were added to black-sided, flat-bottom (optically clear), 96-well microtiter plates containing schistosomula (1000 parasites/well in triplicate) at 10 µM concentrations. Schistosomula were cultured (as already indicated; 37°C, 5% CO_2_) in the presence of each compound for 24 hr before viability levels were assessed.

### Schistosomula viability determination in response to test compounds

After the 24 hr culturing period in the presence of test compounds, and prior to addition of fluorescent dyes, all schistosomula were washed three times to remove test compound and culture media supplements. Each wash consisted of centrifuging microtiter plates containing schistosomula at 100×*g* for 5 min, removal of half the old culture media and replacement with an equal quantity of fresh DMEM (lacking phenol red). After washing the parasites, PI and FDA were simultaneously added to each well of the microtiter plate to obtain a final concentration of 2.0 µg/ml and 0.5 µg/ml respectively.

The 96-well microtiter plates, now containing fluorescently labeled parasites, were subsequently loaded into a BMG Labtech Polarstar Omega plate reader containing appropriate filters for the simultaneous detection of PI (544nm excitation/620nm emission) and FDA (485nm excitation/520nm emission). All fluorescent values were obtained with the plate reader incubator set at 37°C to ensure efficient esterase conversion of FDA to fluorescein within live schistosomula. The plate reader automatically sets the photo multiplier tube (PMT) gain for each fluorescent dye and this may slightly vary between experiments. Inclusion of appropriate control samples (live and dead schistosomula) compensates for any inter-plate variations in gain settings.

### Data handling and statistical analysis

All data were exported into Microsoft Excel for organization and into Minitab (Version 14) for statistical analyses. A One-Way Analysis of Variance (ANOVA) followed by *post hoc* testing with Fisher's least significant difference (LSD) was used to detect statistical differences between treatments. Viability percentages were either: (1) converted into probits for auranofin dose response curve generation and calculation of LD_50_ values or (2) Arcsin transformed in order to stabilize variances prior to the use of appropriate statistical analyses.

Numbers of live and dead schistosomula in each well of a microtiter plate were calculated using the following equations:
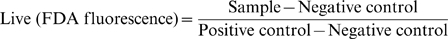


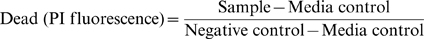
‘Samples’ represent fluorescence intensity units collected from parasites incubated with test compounds. ‘Negative control’ represents fluorescence intensity units collected from parasites killed with 10µM auranofin or heat shock (10 min incubation at 65°C), while the ‘Positive control’ represents fluorescence intensity units collected from untreated parasites. ‘Media control’ represents fluorescence intensity units originating from wells containing only media (no parasites).

To determine schistosomula viability in response to each test compound, a second calculation was employed. This normalization compensated for inter-well variability in schistosomula numbers across the microtiter plate, and to facilitate accurate viability comparisons between test compounds from different microtiter plates:
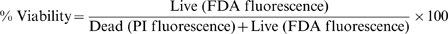



## Results

### Microscope examination of *in vitro* transformed schistosomula

**Figure 1:**
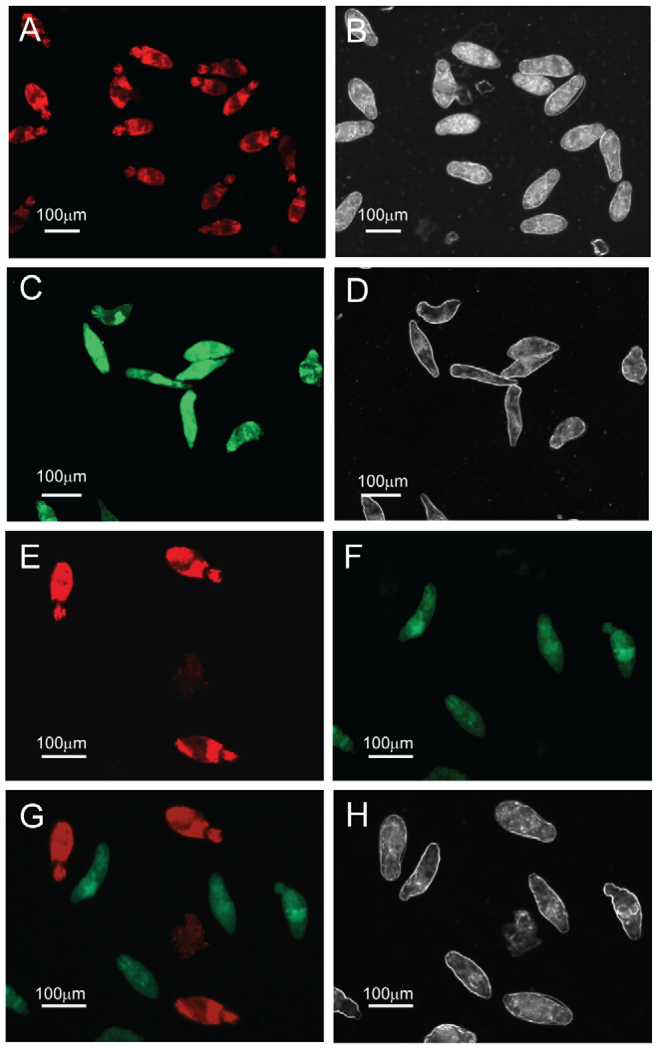
Fluorescein diacetate (FDA) and propidium iodide (PI) can be used to differentially quantify schistosomula viability. Mechanically-transformed schistosomula were prepared, heat-killed (dead) or left untreated (live) and stained with FDA, PI or both fluorophores according to the *Methods*. Epi-fluorescent and plane polarized microscopy was used to visualize uptake of fluorophores and examine schistosomula morphology. (A) Dead schistosomula stained with PI and detected by a rhodamine filter (536 nm), (B) Dead schistosomula visualized by polarized light, (C) Live schistosomula stained with FDA and detected by a FITC filter (494 nm), (D) Live schistosomula visualized by polarized light (E) Mixtures of dead and live schistosomula simultaneously stained with PI/FDA and detected by a rhodamine filter, (F) Mixtures of dead and live schistosomula simultaneously stained with PI/FDA and detected by a FITC filter, (G) Differential detection of PI-positive dead and FDA-positive live schistosomula by superimposition of both 536 nm and 494 nm epifluorescent spectra, (H) Differential morphology of dead and live schistosomula detected by polarized light.

To first determine whether propidium iodide (PI) and fluorescein diacetate (FDA) could be used individually to detect dead or live *in vitro* transformed *S. mansoni* schistosomula, an initial fluorophore uptake experiment was performed with parasite viability assessed by fluorescent microscopy. Here, *in vitro* transformed schistosomula, cultured for 24 hr, were either heat killed at 65°C for 10 min or left undisturbed at physiological conditions (37°C). Heat-killed parasites were then subsequently single-stained with PI (2.0 µg/ml) whereas live parasites were incubated with FDA (0.5 µg/ml). All dead parasites fluoresced (Fig. 1A, 16 out of 16) when visualized for PI uptake at 536 nm, with corresponding polarized bright field microscopy imaging of the same schistosomula samples providing morphological confirmation of death (i.e. parasites that were uniform in shape and size and displayed no movement (Fig. 1B)). Furthermore, all live parasites fluoresced (Fig. 1C, 10 out of 10) when visualized for FDA uptake at 494 nm, with corresponding polarized bright field microscopy imaging of the same schistosomula samples providing visual confirmation of a physiological normal phenotype (i.e. parasites displaying a variety of shapes and sizes as the result of movement during *in vitro* culturing, Fig. 1D). Further quantification of fluorophore uptake was performed on four different regions of slides containing PI stained schistosomula and from five different regions of slides containing FDA stained schistosomula. Here 100% of the parasites were stained with either of the two examined fluorophores (39 out of 39 dead schistosomula were PI positive and 48 out of 48 live schistosomula were FDA positive), confirming the utility of the chosen fluorophores for evenly staining schistosomula (data not shown).

By demonstrating that PI could effectively be used to stain dead schistosomula and FDA was capable of identifying live parasites, a further experiment was conducted to determine whether PI and FDA could simultaneously be used to detect individual live (FDA positive) and dead (PI positive) *in vitro* transformed schistosomula from a mixed population of differentially viable parasites. Here, *in vitro* transformed schistosomula, cultured for 24 hr, were either heat killed at 65°C for 10 min or left undisturbed at physiological conditions (37°C). Equal numbers of live and dead schistosomula were mixed, simultaneously stained with PI (2.0 µg/ml) and FDA (0.5 µg/ml) and examined for the presence or absence of differential fluorophore emission. Whereas heat-killed schistosomula exhibited strong PI staining (minimal FDA staining), physiologically normal schistosomula conversely displayed strong FDA staining (no observable PI staining) (compare Fig. 1E and Fig. 1F). This differential staining of individual live and dead parasites was further supported by observations of undetectable co-localization of PI and FDA signals (within the same cell of an individual schistosomulum, detectable as a yellow signal), schistosomula phenotype and parasite motility as detected by both fluorescent and polarized bright field microscopy (Fig. 1G and 1H).

### Optimization of fluorophore/schistosomula incubation time

**Figure 2:**
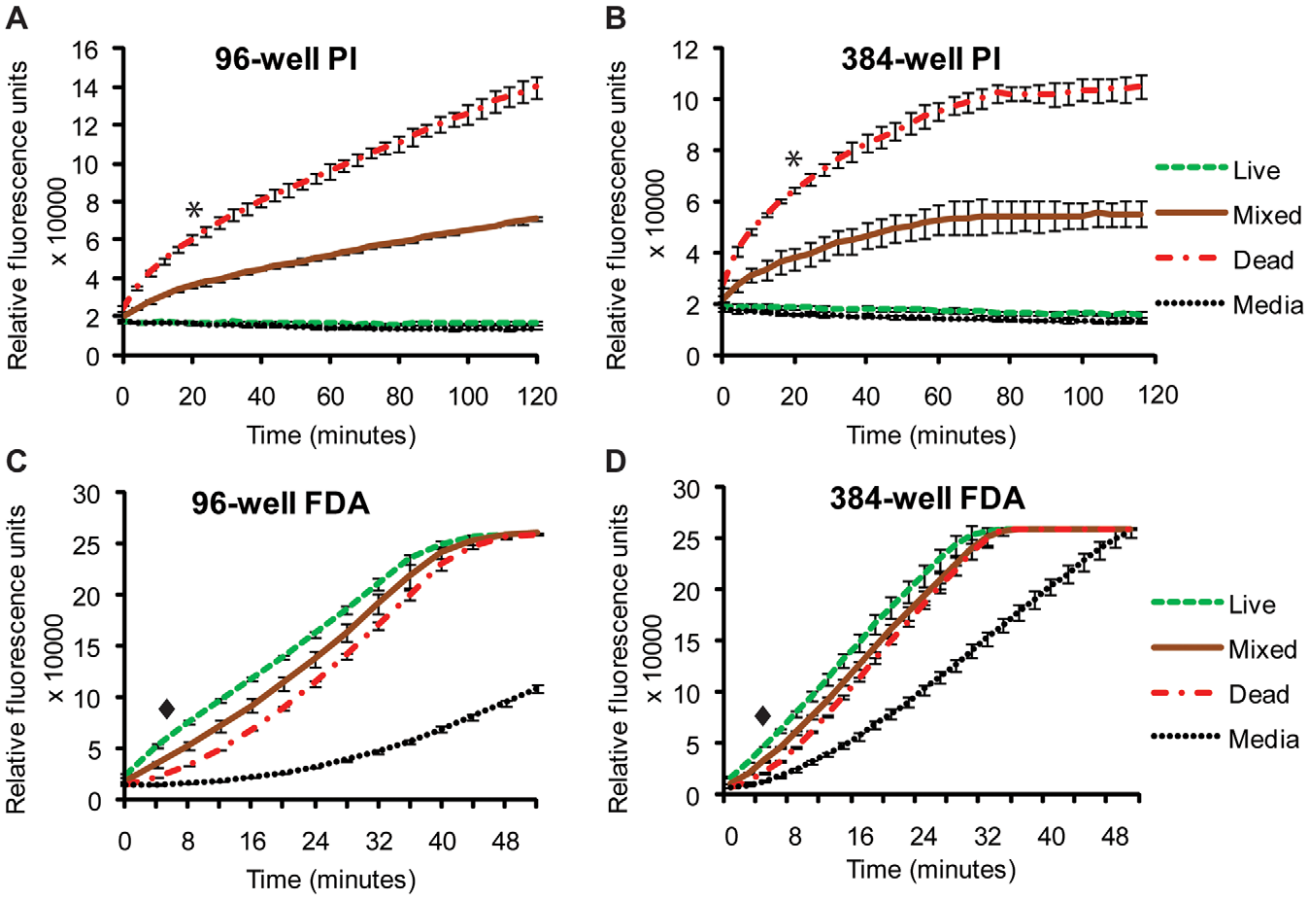
A kinetic study of FDA and PI emission reveals the optimal timeframes to collect fluorescent signals from stained schistosomula samples. Mechanically-transformed schistosomula were cultured for 24 hr, heat killed (dead) or left untreated (live), simultaneously stained with PI and FDA and subjected to fluorescent readings at minute intervals. PI fluorescence (544 nm) was measured over 120 min whereas FDA fluorescence (485 nm) was measured over 51 min. (A) PI fluorescence data collected from a 96-well microtiter plate, (B) PI data collected from a 384-well microtiter plate, (C) FDA data collected from a 96-well microtiter plate and (D) FDA data collected from a 384-well microtiter plate. All fluorescent readings were obtained from a BMG Labtech Polarstar Omega microtiter plate reader. Red lines represent fluorescent data originating from wells containing dead schistosomula, green lines represent fluorescent data originating from wells containing live schistosomula, brown lines represent fluorescent data from wells containing an equal number (mixed) of dead and live schistosomula and dotted lines represent fluorescent data originating from wells containing no schistosomula (media). * Indicates chosen time point for collecting PI data and ♦ indicates chosen time point for collecting FDA data in subsequent experiments. All experiments were performed at least three times. Schistosomula were plated at 1000 parasites/well in the 96-well microtiter plate format and 200 parasites/well in the 384-well microtiter plate format.

A critical first parameter in translating these observations of differential PI and FDA staining of schistosomula to a method that takes advantage of the high-throughput potential of a microtiter plate assay (in both 96- and 384- well formats) was to determine the optimal timeframe that each fluorescent dye should be incubated with parasite samples to insure maximal reproducible detection of viability (Fig. 2). Here, *in vitro* transformed schistosomula, cultured for 24 hr, were either killed by heat shock (65°C for 10 min) or left unaltered at physiological culturing conditions (37°C). Three samples were subsequently derived from these schistosomula cultures, which included physiologically normal, untreated parasites (Fig. 2, green line), heat-killed parasites (Fig. 2, red line) and an equal mixture of physiologically normal and heat-killed parasites (Fig. 2, brown line). Derived schistosomula samples (in triplicate) were co-stained with PI (2.0 µg/ml) and FDA (0.5 µg/ml) and subjected to fluorescent intensity measurements (BMG Labtech Polarstar plate reader) every minute for 120 min. Fluorescent intensity values derived from triplicate wells containing DMEM (lacking phenol red), PI (2.0 µg/ml) and FDA (0.5 µg/ml) were also obtained over this timeframe (Fig. 2, dotted line).

Throughout the assayed 120 min timeframe, the three tested schistosomula samples (regardless of plate format) demonstrated clear differences in PI fluorescence emission, with the dead parasites demonstrating the highest PI values, the live parasites demonstrating the lowest PI values and the mixed population of live and dead parasites demonstrating intermediate PI values between the extremes (Figs. 2A and 2B). While statistical significance in measured PI fluorescent emission continued to increase (i.e. *p*<0.05 at 1 min and *p*<0.001 for any time point after 4 min) with time for any of the compared schistosomula samples, PI fluorescent emission differences between physiologically normal schistosomula (live) and DMEM (lacking phenol red) samples remained small throughout the duration of the 120-minute timeframe and never reached statistical significance (*p*<0.001). Therefore, based on these analyses, the optimal PI incubation time to distinguish between dead and live schistosomula is between 4 and 120 min, regardless of plate format. We chose 20 min to collect PI data as it provided an adequate time window to process multiple microtiter plates (indicated in Fig. 2A and 2B) and was well within the calculated window of accurately being able to determine statistical significance amongst live and dead schistosomula.

Whereas PI staining of parasite samples yielded differential fluorescent results over the entire 120-minute timeframe (Fig. 2A and 2B), FDA staining of live, dead and mixed schistosomula populations in either 96-well or 384-well microtiter plate formats generated fluorescent data that quickly reached a plateau (51 min for 96-well microtiter plates, Fig. 2C and 32 min for 384-well microtiter plates, Fig. 2D). Therefore, collection of FDA fluorescence was halted at 51 min. Nonetheless, FDA fluorescent intensity units measured across these time intervals produced data as expected with live parasites fluorescing brightest, dead parasites weakest and mixed live/dead parasite populations intermediate between the two (Fig. 2C and 2D). Furthermore, differences in detected FDA fluorescence between any of the three schistosomula samples (in both microtiter plate formats) were found to be statistically significant between 3 min and 12 min. However, unlike the PI timecourse (Fig. 2A and 2B), differences in FDA fluorescent emission originating from wells containing DMEM and all schistosomula samples were statistically significant beginning at 3 minutes (*p*<0.05). This finding makes DMEM unsuitable for use as a blank for detecting non-specific FDA fluorescence. Therefore, based on these analyses, the optimal FDA incubation time to distinguish between dead and live schistosomula in both microtiter plate formats is between 3 and 12 min. We chose 5 min (indicated in Fig. 2C and 2D) to collect FDA data in both microtiter plate formats as this fluorophore is prone to spontaneous hydrolysis [15].

### Determination of assay sensitivity

**Figure 3 pntd-0000759-g003:**
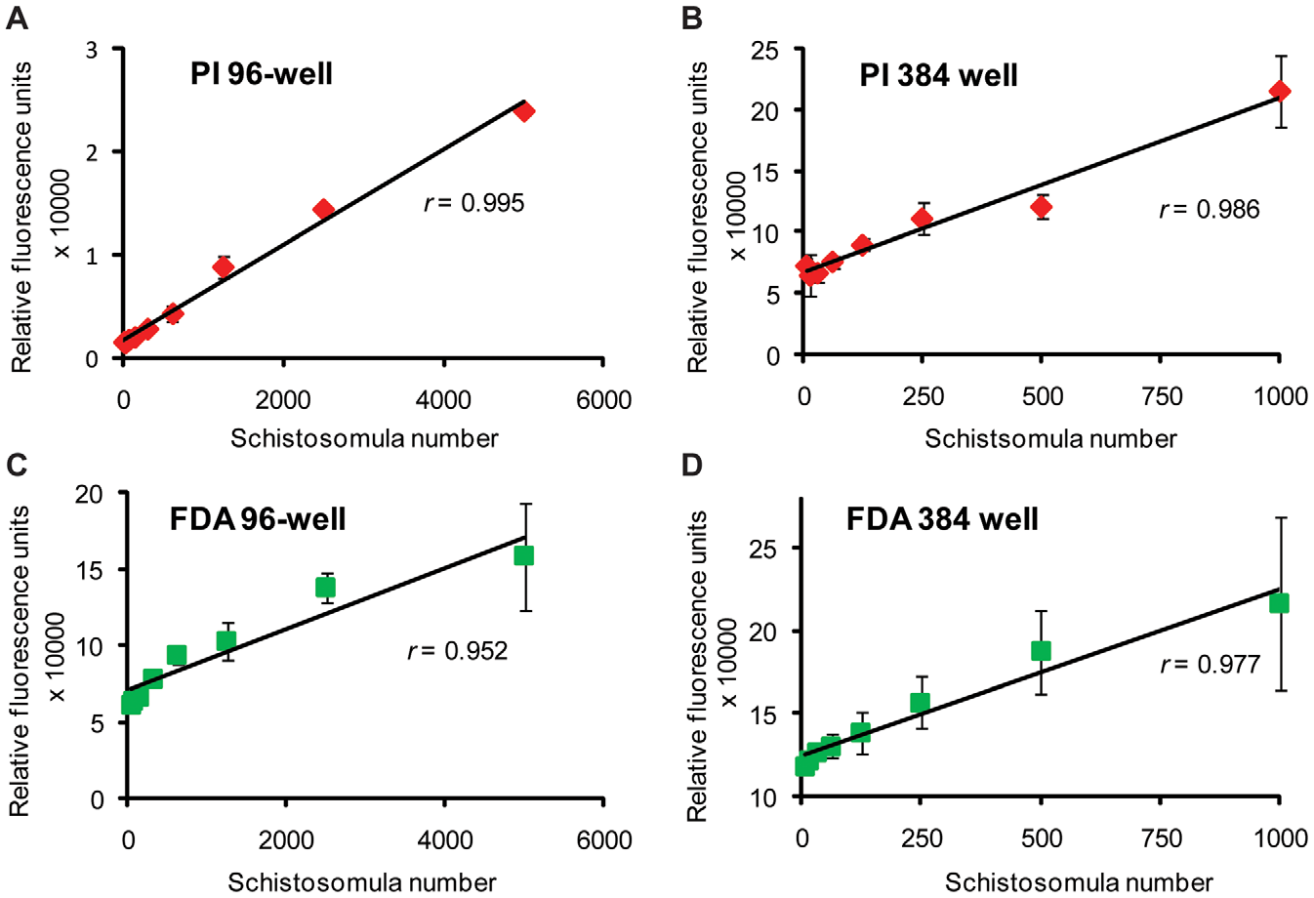
A schistosomula titration series reveals the optimal number of parasites to be used for PI and FDA fluorescent detection in both 96-well and 384-well microtiter plates. Mechanically-transformed schistosomula were cultured for 24 hr, heat killed (dead) or left untreated (live) and stained with either PI (dead parasites) or FDA (live parasites). (A) Dead schistosomula were titrated from 5000 to 36 parasites per well (in triplicate) in a 96-well microtiter plate and PI fluorescence (544 nm) obtained at 20 min, (B) Dead schistosomula were titrated from 1000 to 8 parasites per well (in triplicate) in a 384-well microtiter plate and PI fluorescence (544 nm) obtained at 20 min, (C) Untreated, live schistosomula were titrated from 5000 to 36 parasites per well (in triplicate) in a 96-well microtiter plate and FDA fluorescence (485 nm) measured at 5 min, (D) Untreated, live schistosomula were titrated from 1000 to 8 parasites per well (in triplicate) in a 384-well microtiter plate and FDA fluorescence (485 nm) measured at 5 min. All fluorescent readings were collected from a BMG Labtech Polarstar Omega microtiter plate reader. These results are representative of two independent experiments.

**Figure 4:**
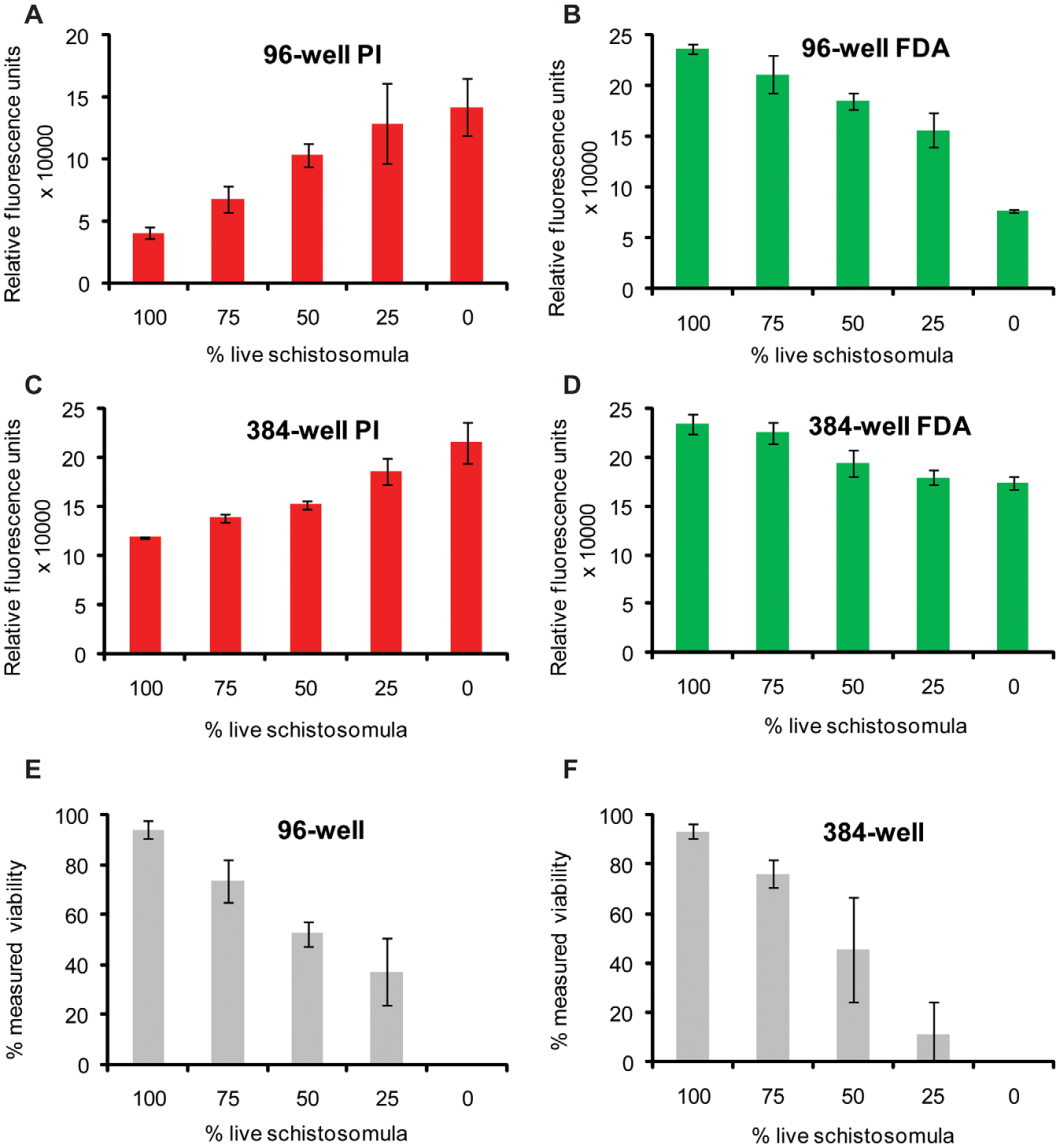
Dual FDA and PI staining of schistosomula samples allows for fluorescent quantification of parasite viability. Mechanically-transformed schistosomula were cultured for 24 hr, heat killed (dead) or left untreated (live), distributed into wells at pre-defined percentages (100% live, 75% live, 50% live, 25% live 0% live) and simultaneously stained with PI and FDA. (A) Pre-defined percentages of live and dead schistosomula (1000 parasites per well, in triplicate) distributed into a 96-well microtiter plate with PI fluorescence (544 nm) measured at 20 min, (B) Pre-defined percentages of live and dead schistosomula (1000 parasites per well, in triplicate) distributed into a 96-well microtiter plate with FDA fluorescence (485 nm) measured at 5 min, (C) Pre-defined percentages of live and dead schistosomula (200 parasites per well, in triplicate) distributed into a 384-well microtiter plate with PI fluorescence (544 nm) detected at 20 min, (D) Pre-defined percentages of live and dead schistosomula (200 parasites per well, in triplicate) distributed into a 384-well microtiter plate with FDA fluorescence (485 nm) detected at 5 min. (E) Schistosomula viability calculated from fluorescent data displayed in A and B, (F) Schistosomula viability calculated from fluorescent data presented in C and D. Formula for viability calculations is indicated in the *Methods* section. These results are representative of experiments performed three times.

To determine the sensitivity limits of this fluorescence-based assay in measuring schistosomula viability in a medium and a high throughput manner, two different experimental approaches were considered (Figs. 3 and 4). In the first approach (Fig. 3A–D), schistosomula were serially diluted (5000 reducing to 36 parasites for a medium throughput 96-well plate format; 1000 reducing to 8 parasites for a high-throughput 384-well plate format, in triplicate) to identify the absolute minimum number of parasites that could be reproducibly detected. Briefly, *in vitro* transformed schistosomula, cultured for 24 hr, were either heat killed (65°C for 10 min) or left unaltered at physiological conditions (37°C). After serially diluting these differentially treated schistosomula samples, FDA or PI fluorophores were added and measurement of fluorescence intensity proceeded, within the parameters determined above (5 min for FDA and 20 min for PI).

Correlation analysis revealed a strong linear relationship between PI fluorescence and dead schistosomula number. For the 96-well plate format (Fig. 3A) a high correlation (*r* = 0.995) existed between the measured PI fluorescence and numbers of dead schistosomula sampled, while for the 384-well plates (Fig. 3B) a slightly lower correlation (*r* = 0.986) existed between measured PI fluorescence and numbers of dead schistosomula.

Correlation analysis of FDA fluorescence and live schistosomula also revealed a strong linear relationship (*r* = 0.952 for the 96-well plate format, Fig. 3C and *r* = 0.977 for the 384-well format, Fig. 3D), although the linearity was affected by an FDA-associated fluorescence plateau effect seen when increased numbers of schistosomula were assayed. From these experiments, it was determined that optimal numbers of schistosomula for assays conducted in a 96-well microtiter plate are between 500 and 2500 parasites per well, while for assays conducted in a 384-well microtiter plate, optimal numbers of schistosomula are between 50 and 500 per well.

The second approach used to interrogate the sensitivity of this fluorescence-based assay for detecting schistosomula viability was to replicate conditions where varying percentages of live and dead schistosomula would be found in the same sample (i.e. *in vitro* drug assays, where the drug tested is less than 100% efficient in killing). By mixing different percentages of live (physiologically normal) and dead (heat killed) parasites (Fig. 4), we tested the ability of PI (Figs. 4A and 4C) and FDA (Figs. 4B and 4D) to distinguish viability in a population of 1000 schistosomula in a 96-well plate (Fig. 4E) and 200 schistosomula in a 384-well plate (Fig. 4F). In both plate formats, intra-well PI fluorescence decreased and FDA fluorescence increased when greater percentages of live schistosomula were being examined. This made it possible to differentially identify schistosomula viability in intervals of 25% for both 96- and 384-well plate formats. However, the standard deviations reported for some values indicated that there was variability inherent in pipetting with discrete units of this size. The use of a dual staining method was, therefore, important for counteracting any pipetting errors and verifying that differences in fluorescence are genuinely caused by differences in mortality.

### Validation of the dual fluorescent assay for identifying therapeutic lead compounds

**Figure 5:**
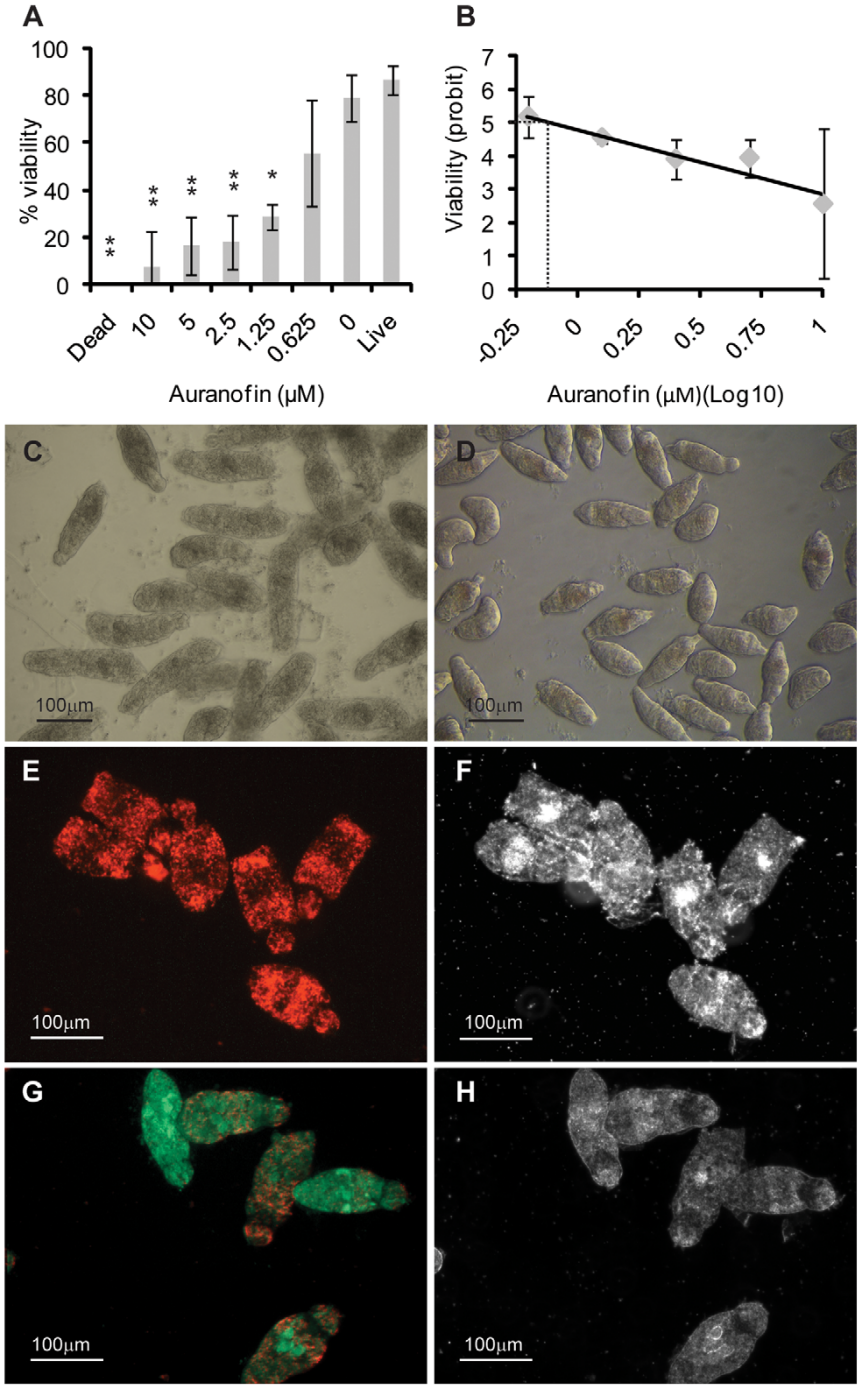
Dual-fluorescence viability determination of auranofin treated schistosomula. Mechanically-transformed schistosomula were cultured for 24 hr, incubated with different concentrations of auranofin for an additional 24 hr, washed and subsequently co-stained with both PI and FDA. PI- (544 nm, collected at 20 min) and FDA- (485 nm, collected at 5 min) fluorescence intensity units were converted into viability measures according to the formula described in the *Methods*. (A) Dose-dependent anti-schistosomula effect of auranofin as indicated by % viability. Treatments showing statistically significant differences in viability when compared to untreated (live) schistosomula are indicated with * (*p*<0.05) or ** (*p*<0.001). (B) Auranofin dose-response curve by which an LD_50_ was calculated by plotting the probit transformation of the % viability to the Log_10_ transformation of auranofin concentration. Dotted line indicates the average LD_50_ value calculated from three replicates. (C) Light microscope image of schistosomula treated with 10µM auranofin for 24 hr. (D) Light microscope image of untreated schistosomula. (E) Epi-fluorescent and (F) plane polarized light micrograph of schistosomula treated with 10µM auranofin for 24 hr, then incubated with both PI and FDA. (G) Epi-fluorescent and (H) plane polarized light micrograph of schistosomula treated with 1µM auranofin for 24 hr, then incubated with PI and FDA. ‘Dead’ represents schistosomula killed by heat-treatment and ‘0 µM’ auranofin represents schistosomula incubated with 1% (v/v) DMSO (auranofin solvent). These results are representative of experiments performed three times.

To validate this fluorescent-based viability assay for practical application in medium to high-throughput drug screening, it was used to assess the efficacy of a known anti-schistosomula compound, auranofin (Fig. 5). An inhibitor of parasite thioredoxin glutathione reductase (TGR), auranofin has been shown (estimated by light microscopy examination) to be 100% lethal to *in vitro* cultured, mechanically-transformed schistosomula at 10 µM concentrations, 24 hr after treatment [9].

In agreement with published reports, there is a clear and titratable anti-schistosomula effect mediated by auranofin with the dual fluorescent staining procedure allowing viability quantification of each drug concentration at 24 hours post-treatment (Fig. 5A). Percent viability transformations into probit values also allowed an auranofin LD_50_ of 0.82±0.49 to be calculated (Fig. 5B). Maximum drug effect was seen at 10 µM, where microscopic examination of schistosomula confirmed that death was 100% (Fig. 5C, 5E and 5F), when compared to untreated parasites (Fig. 5D). At an auranofin concentration within the calculated LD_50_ range (1 µM), schistosomula contained cells either labeled with PI or FDA throughout the lophotrochozoan body (Fig. 5G and 5H). Statistically-significant, auranofin-mediated mortality, compared to either vehicle treated (DMSO) schistosomula or untreated parasites (live), was also observed for drug concentrations of 5 µM, 2.5 µM and 1.25 µM.

**Figure 6:**
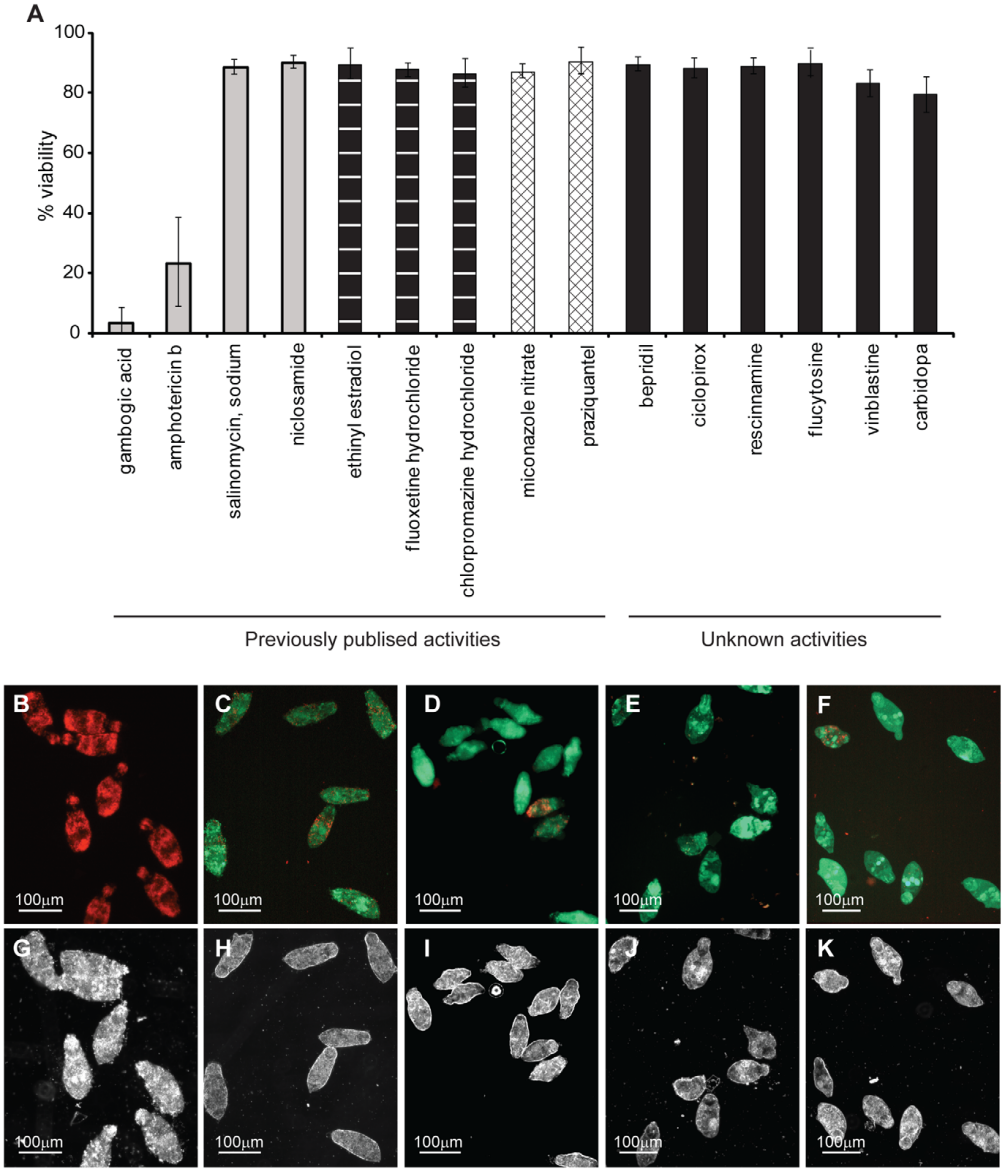
Application of the dual-fluorescent viability assay for determining the anti-schistosomula effect of selected compounds with previously-described or unknown activities. Mechanically-transformed schistosomula were cultured for 24 hr, incubated with compounds (10 µM) for an additional 24 hr, washed and subsequently co-stained with both PI and FDA. PI- (544 nm, collected at 20 min) and FDA- (485 nm, collected at 5 min) fluorescence intensity units were converted into viability measures according to the formula described in the *Methods*. (A) Calculated schistosomula viability in response to each compound tested (further details can be found in Dataset S1). Representative epi-fluorescent and plane polarized microscope images of schistosomula treated with (B and G) gambogic acid, (C and H) sodium salinomycin, (D and I) niclosamide, (E and J) praziquantel and (F and K) ciclopirox are indicated. Compounds designated as having ‘previously published activities’ (grey histograms – death, vertical lines within histograms – overactive, hatched lines within histograms – rounded) were selected from those described by Abdulla *et al.* [10] while those compounds indicated as having ‘unknown activities’ (black histograms) were selected from Berriman *et al.* [3]. These results are representative of two independent experiments.

Further validation of this viability assay was next performed by assessing the effect of compounds with previously-identified [10] or suggested anti-schistosomal activities [3] (Fig. 6). These compounds included four (gambogic acid, sodium salinomycin, niclosamide nitrate and amphotericin b) that have been described to induce schistosomula mortality [10], three (ethinyl estradiol, fluoxetine hydrochloride and chlorpromazine hydrochloride) that have been reported to induce schistosomula over-activity [10], two (miconazole nitrate and praziquantel) that have been shown to produce a shape alteration (‘rounded’ phenotype) [10] and six (bepridil, ciclopirox, rescinnamine, flucytosine, vinblastine and carbidopa) that have never been tested on schistosomula [3] (full details in Dataset S1). Of the tested compounds with previously-recorded effects on schistosome phenotypes [10], our dual-fluorescent viability screen, reassuringly showed broad agreement (Fig. 6A). Here, the compounds previously described as inducing an over-active (ethinyl estradiol, fluoxetine hydrochloride and chlorpromazine hydrochloride) or rounded (miconazole nitrate and praziquantel) phenotype, as expected, did not affect schistosomula viability. Fluorescent readings obtained from wells containing parasites treated with these compounds were no different from wells containing untreated parasites (average viability 84.3%, data not shown). Microscopic examination of these treated parasites confirmed their viability (e.g. Fig. 6E and Figure S1). However, only two of four previously defined anti-schistosomula compounds (gambogic acid and amphotericin b) induced measurable death as determined by our dual-fluorescent viability assay (confirmed by microscopic examination of schistosomula, e.g. Fig. 6B and Fig. S1). Sodium salinomycin and niclosamide produced fluorescent viability measurements similar to those derived from untreated parasites. Detailed microscopic examination of schistosomula treated with sodium salinomycin demonstrated that the parasites were strongly FDA positive (with some PI positive cells) (Fig. 6C), dark and granular (Fig. 6H and Figure S1, panel B), but motile (Figure S1, panel B) whereas niclosamide treated schistosomula appeared morphologically (Fig. 6I and Figure S1, panel H) and fluorescently (Fig. 6D) similar to praziquantel treated parasites (rounded phenotype, Fig. 6E, Fig. 6J and Figure S1, panel I). Of the six compounds with an unknown, but suggested, anti-schistosome effect, none showed strong decreases in viability as determined by the dual fluorescence assay or epifluorescent microscopy (e.g. ciclopirox, Fig. 6F) under the conditions tested in this study. However, upon further microscopic examination, some compounds did induce shape alterations in schistosomula phenotype (e.g. ciclopirox, Fig. 6K and Figure S1, panel K).

## Discussion

In this study we detail the development and validation of a novel medium- to high- throughput microtiter assay for assessing viability in the schistosomula stage of the human parasite *S. mansoni*. Infection with this parasitic trematode leads to chronic pathology in endemic populations accounting for disability adjusted life year (DALYs) estimates surpassing malaria or tuberculosis [16], and is responsible for approximately 200,000 deaths per annum [2]. With only one drug (praziquantel) predominantly used to treat this disease, the potential for drug resistant parasite strains poses a real threat to global control initiatives [17] and supports the vital need for identifying novel therapeutic drug classes or bioactive molecules. To date, high-throughput discovery of new and effective anti-schistosomal compounds has been hampered by the lack of a uniform and quantifiable evaluation method, with current assessment of trematode viability involving subjective and labor-intensive microscopic examination [7]. Application of improved and objective methods in detecting schistosome viability will, therefore, greatly enhance the discovery of next generation chemotherapeutic agents. Here, we provide evidence that a dual-fluorescent, whole schistosomula bioassay (helminth fluorescent bioassay; HFB) contains many criteria required for successful high-throughput screening and demonstrate an easily-adaptable methodology by which the *Schistosoma* genomes [3], [18] can be rapidly screened for urgently needed drug targets.

While many fluorophores are available for use in viability measurements, propidium iodide (PI) and fluorescein diacetate (FDA) were initially chosen due to their distinct wavelength emission profiles, wide availability from suppliers and low cost. These criteria and the experimental validation that individual schistosomula of polarized viability (either dead or alive) only fluoresced red (PI positive or dead) or green (FDA positive or alive) and not yellow (Fig. 1), indicated that these two fluorophores stained schistosomula cells independently and could inexpensively be used to quantify parasite viability, when applied to methodology involving a microtiter plate reader equipped to detect fluorescence. While variation in fluorophore (both PI and FDA) uptake was noticeable amongst individual schistosomula (Fig. 1), this was expected due to the non-synchronous nature of schistosome development *in vitro* as well as experimental factors that commonly affect epifluorescent microscopy including focal point differences between individuals, imperfections in the glass coverslip, localization of a parasite near an air bubble or digital processing of captured images. Importantly, these factors did not interfere with the acquisition of highly-reproducible fluorescence collected from a population of assayed schistosomula when measured by a plate reader (i.e. Figs. 2–4). Taken together, PI and FDA contained multiple characteristics well-suited for detection of schistosomula viability and, therefore, adapting these fluorophores to microtiter plate formatting was pursued.

While minor differences existed between plate formats (Figs. 2, 3 and 4A–D; 96- versus 384-well), these did not affect the overall quantification of schistosomula viability (Fig. 4E vs 4F). For example, due to smaller surface areas within wells of a 384-well microtiter plate (compared to 96-well microtiter plates), fluorescent intensity slopes (when correlated to parasite number, Fig. 3 or parasite viability, Fig. 4A–D) were not as steep when compared to fluorescent intensity measurements collected from 96-well microtiter plates. Nevertheless, strong correlation coefficients, comparing fluorescent intensity units with schistosomula number (Fig. 3), were obtained in both microtiter plate formats. From these experiments, it was also apparent that FDA could be subjected to spontaneous hydrolysis (Fig. 2C and 2D). While this has been documented before in other systems [15], and could be due to media components or residual esterase activity released by the dead schistosomes, the fluorescence measured between live and dead parasites were sufficiently large to detect a significant difference (between 3 min and 12 in both 96-well and 384-well plate formats). Nevertheless, substitution fluorophores for FDA, including calcein AM and sulfofluorescein diacetate, are being investigated in current optimizations to the HFB.

The observation that calculated, intra-well schistosomula viability (mathematically derived from plate reader measurements of both PI and FDA, Fig. 4A–D) paralleled the proportion of viable schistosomula dispensed into each well (Fig. 4E and 4F), demonstrated that FDA and PI could cooperatively be used to sensitively and accurately differentiate amongst percentages of viable parasite populations within either 96- or 384- well microtiter plate formats. This is essential as any high-throughput anthelmintics screening assay may include compounds that do not induce 100% schistosomula lethality and, therefore, use of both PI and FDA allow for accurate quantification across a range of viability endpoints (between 0% viable to 100% viable).

Taking advantage of PI and FDA's cooperative ability to differentially stain schistosomula and the capability of a microtiter plate reader to sensitively quantify inter-well parasite viability, we applied the HFB to compounds previously shown to affect schistosomula phenotype and survival [9], [10]. The first compound selected was auranofin, a potent thioredoxin glutathione reductase inhibitor that has previously been shown to kill larval schistosomula and adult schistosomes [9]. While our HFB confirmed that auranofin does induce schistosomula death (Fig. 5), our results additionally demonstrated a much finer sensitivity in detecting auranofin LD_50_ levels (concentration of auranofin that kills 50% of the assayed biological material). Kuntz *et al.* [9], using microscopy, reported LD_50_ levels of auranofin on schistosomula to be between 2 µM and 5 µM. Our auranofin titration series, detected by fluorescent-based microtiter plate quantification, demonstrated that this compound has an anti-schistosomula LD_50_ activity of 0.82 µM±0.49 µM. This objective determination is approximately 4 fold more sensitive than what was previously published and clearly demonstrates the increased sensitivity of the HFB and a standardized methodology. Interestingly, schistosomula, treated with 1 µM auranofin (within the calculated LD_50_ range) and visualized by epifluorescent microscopy, displayed cells that were stained with either PI or FDA throughout the body (Fig. 5G). This finding clearly indicates that the HFB is sensitive enough to detect compound-induced changes in fluorescence affecting different cell populations within individual schistosomula as well as between schistosomula and may be useful in identifying compounds that induce cellular stress (in addition to whole organism viability).

We next expanded use of the HFB to verify the activity of selected compounds on schistosome phenotype and viability as previously demonstrated by Abdulla *et al.* [10]. Two of the nine compounds screened showed variance with the published results. The first of these, sodium salinomycin, in contrast to previously published observations [10], did not induce schistosomula lethality when measured by the HFB or epifluorescent microscopy (Fig. 6A and 6C). The other compound tested that gave conflicting results to that which were published is niclosamide. Niclosamide is recorded in the literature as an anthelmintic, however, its use as such is mainly against cestodes [19] with anti-trematode activity only being reliably recorded against immature *Paramphistomum* spp. [20]. Its use in schistosomiasis control seems to be limited as a moluscicide for control of the intermediate snail host (e.g [21]). While both sodium salinomycin and niclosamide treated schistosomula were stressed (decrease in motility - sodium salinomycin, Fig. 6H; shape alterations – niclosamide, Fig. 6I; some PI positive cells – both compounds, Figs. 6C and 6D), it is not clear why our results differ from Abdulla *et al.* [10] in relationship to measured viability. A likely explanation may be associated with how the schistosomula were processed before drug treatment in the two studies. While our study utilized schistosomula, cultured at 37°C, 24 hr after mechanical transformation, Abdulla *et al.* used schistosomula, maintained on ice, 2 hr after mechanical transformation [10]. We propose that schistosomula, maintained at 4°C, are likely to be more physiologically stressed than parasites cultured at 37°C and, therefore, more susceptible to additional manipulations such as drug treatment. These differences in schistosomula processing, as well as the fact that alternative solvents for niclosamide and sodium salinomycin were used (ethanol and acetone in this study, whereas DMSO in Abdulla *et al.* [10]), could likely explain the observed differences.

The final application of the HFB for measuring schistosomula viability was directed towards the verification of potential novel anthelmintics from those identified by the chemogenomics screening strategy outlined in the recent publication of the *S. mansoni* genome [3]. Using an iterative bioinformatics approach, Berriman *et al.* identified 26 *S. mansoni* target proteins that have strong orthology to human proteins currently being targeted by approved drugs [3]. It was suggested that ‘drug-repositioning’ strategies that re-use existing drugs offer potential savings and cost benefits to the development of novel anthelmintics, especially in the context of neglected tropical diseases like schistosomiasis. Therefore, six drugs with activity against human proteins (bepridil - calmodulin, ciclopirox - deoxyhypusine synthase, rescinnamine – vesicular amine transporter, flucytosine – bifunctional dihydrofolate reductase-thymidylate synthase, vinblastine – beta tubulin and carbidopa – aromatic amino acid decarboxylase) were tested against the *Schistosoma* orthologs (Smp_026560, Smp_065120, Smp_121920, Smp_135460, Smp_030730 and Smp_171580 respectively) using the HFB (Fig. 6A). While none of the selected drugs showed any notable effect on schistosomula viability (at 10 µM), some induced phenotypic changes in parasite morphology (Fig. 6K and S1) and caused a degree of physiological stress (some PI positive cells, Fig. 6F). These compounds, like sodium salinomycin and niclosamide (see above) warrant further investigations. The most likely explanation for lack of any detectable anthelmintic activity is that the tested drugs could not efficiently cross the heptalaminate membrane covering the newly transformed schistosomula or that the target proteins were not highly expressed in this larval life stage. Efforts to increase the membrane permeability of all tested drugs (i.e. standardized solubilization in DMSO) are currently being implemented in current screens of schistosomula viability, which likely will decrease this potential confounding factor.

Recently, both Smp_121920 (vesicular amine transporter) and Smp_135460 (bifunctional dihydrofolate reductase-thymidylate synthase) were found to be minimally expressed in the schistosomula lifecycle stage as determined by DNA microarray analysis of the parasite lifecycle [22]. These gene expression results could explain the ineffective anti-schistosomula nature of rescinnamine and flucytosine. However, in the same study [22], Smp_026560 (calmodulin) was abundantly found in newly-transformed schistosomula. This finding, supported by proteomic analyses of schistosomula [23], [24] indicates that calmodulin would be in sufficient quantities for targeting by bepridil. Therefore, although presence of schistosomula targets is necessary for measuring drug effectiveness, it is not sufficient. Other factors (e.g. drug biotransformation and drug synergy) are equally important and should be carefully considered during the interpretation of anti-schistosomula drug screening results.

In summary, we report a methodology that enables the objective measurement of schistosomula viability, which is quantitative, fast and inexpensive. Although we have restricted its application to drug assays, the adapted methodology has the potential to be used for assessing viability during high-throughput RNAi screens and general manipulations of schistosome development. With this assay functioning in both 96- or 384-well microtiter plate formats, it can be readily adapted for use in academic or industrial settings, in particular if robotics or semi-automation were available. Further refinements of the assay could be proposed to increase processivity and facilitate adaptation to other parasitic and non-parasitic worm species. As well as other worm species, the HFB should also be applicable to other *Schistosoma* lifecycle stages, including the adult and this enhancement is among other modifications currently being pursued. As death is not the only criterion that could be measured when assessing potential anthelmintic compounds (compounds that induce stress, paralysis or shape alterations are equally likely to be potent; e.g. niclosamide, sodium salinomycin, ciclopirox, etc.), we are currently integrating phenomics, metabolomics and high content screening approaches to add value to our HFB. The combined use of these multiple and quantifiable methods to describe a compound's effect on helminth development will exponentially increase the number of preliminary hits translated into effective and novel anthelmintic chemotherapies.

## Supporting Information

Dataset S1 Further description of selected compounds used in the dual-fluorescent helminth viability assay.
(0.01 MB XLS)

Figure S1 Light microscope images of schistosomula treated with each of the test compounds. Mechanically-transformed schistosomula were cultured for 24 hr, incubated with compounds (10 µM) for an additional 24 hr, washed and photographed according to Methods. (A) Gambogic acid, (B) Sodium Salinomycin, (C) Ethinyl estradiol, (D) Fluoxetine hydrochloride, (E) Miconazole nitrate, (F) Chlorpromazine hydrochloride, (G) Amphiotericin B, (H) Niclosamide, (I) Praziquantel, (J) Bepridil, (K) Ciclopirox, (L) Rescinnamine, (M) Flucytosine, (N) Vinblastine, (O) Carbidopa.
(7.61 MB TIF)
