# Supplementary figures and images for: Development and Validation of a Quantitative, High-Throughput, Fluorescent-Based Bioassay to Detect Schistosoma Viability
Source: PLoS Negl Trop Dis. 2010 Jul 27;4(7):e759. doi: 10.1371/journal.pntd.0000759 (PMC2910722; doi:10.1371/journal.pntd.0000759)

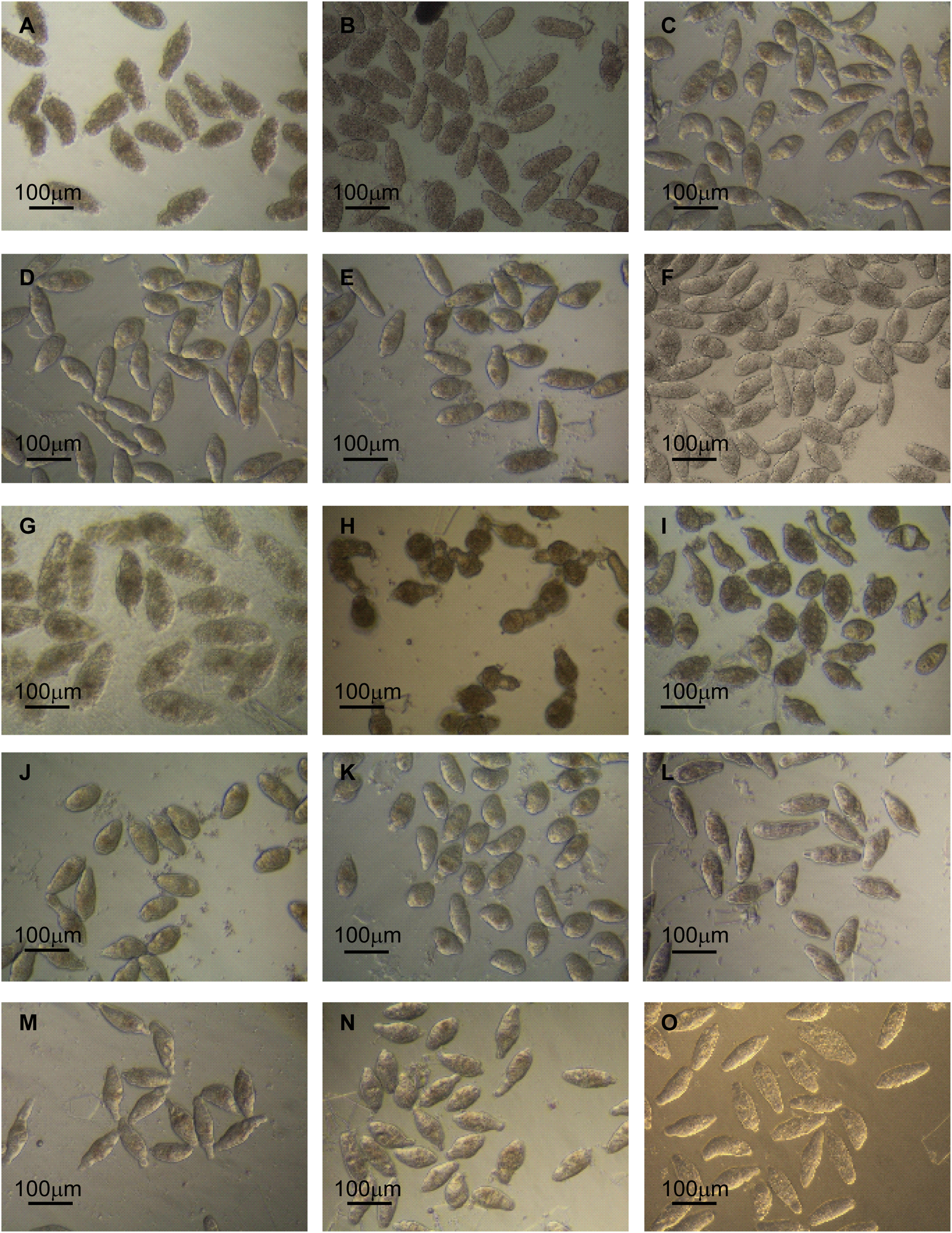

Supplement: Figure S1 — Light microscope images of schistosomula treated with each of the test compounds. Mechanically-transformed schistosomula were cultured for 24 hr, incubated with compounds (10 µM) for an additional 24 hr, washed and photographed according to Methods. (A) Gambogic acid, (B) Sodium Salinomycin, (C) Ethinyl estradiol, (D) Fluoxetine hydrochloride, (E) Miconazole nitrate, (F) Chlorpromazine hydrochloride, (G) Amphiotericin B, (H) Niclosamide, (I) Praziquantel, (J) Bepridil, (K) Ciclopirox, (L) Rescinnamine, (M) Flucytosine, (N) Vinblastine, (O) Carbidopa. (7.61 MB TIF) [file pntd.0000759.s002.tif]
